# Fat Meat

**Published:** 1873-02

**Authors:** 


### FAT MEAT.

High medical authority states that—

1. Of all the persons between the ages of fifteen and twenty-two years, more than one-fifth eat no fat meat.

2. Of persons at the age of forty-five, all, excepting less than one in fifty, habitually use fat meat.

3. Of persons who, between the ages of 15 and 22, avoid fat meat, a few acquire an appetite for it and live to a good old age, while the greater portion die with phthisis before 35.

4. Of persons dying with phthisis, between the ages of 12 and 45, nine-tenths at least have never used fat meat.

The only wise use to be made of these assertions, taking it for granted that they are true, is to eat fat meat if you want it, and if you do not, let it alone. Using fat meat never cured a man of consumption or prevented his having it; its mere use cannot possibly of itself cause consumption because consumption is always caused by poor blood—that is, blood which has not all its healthful constituents, and which, in addition, contains some which do not naturally belong to it; blood, to be rich, health-giving, must contain its natural share of nutritive elements. There is no nutrition in fat or oil of any kind. All the curative agency which oils can have in consumption is in the direction of arresting it in some of its forms. What millions of persons have swallowed gallons of cod liver oil while laboring under consumption, and died for all that. The great general rule in eating is: Take nothing against your inclination, eat in moderation what you like best, but if it is uniformly followed by some discomfort let it alone for the present.

